# Review of Recent Progress of Plasmonic Materials and Nano-Structures for Surface-Enhanced Raman Scattering

**DOI:** 10.3390/ma8063024

**Published:** 2015-05-28

**Authors:** Alan X. Wang, Xianming Kong

**Affiliations:** 1 School of Electrical Engineering and Computer Science, Oregon State University, Corvallis, OR 97331, USA; 2 Department of Forest Products Technology, School of Chemical Technology, Aalto University, P.O. Box 16300, FI-00076 Aalto, Finland; E-Mail: xianmingk@gmail.com

**Keywords:** surface plasmons, plasmonic materials, photonic crystals, surface-enhanced Raman scattering, optical sensors

## Abstract

Surface-enhanced Raman scattering (SERS) has demonstrated single-molecule sensitivity and is becoming intensively investigated due to its significant potential in chemical and biomedical applications. SERS sensing is highly dependent on the substrate, where excitation of the localized surface plasmons (LSPs) enhances the Raman scattering signals of proximate analyte molecules. This paper reviews research progress of SERS substrates based on both plasmonic materials and nano-photonic structures. We first discuss basic plasmonic materials, such as metallic nanoparticles and nano-rods prepared by conventional bottom-up chemical synthesis processes. Then, we review rationally-designed plasmonic nano-structures created by top-down approaches or fine-controlled synthesis with high-density hot-spots to provide large SERS enhancement factors (EFs). Finally, we discuss the research progress of hybrid SERS substrates through the integration of plasmonic nano-structures with other nano-photonic devices, such as photonic crystals, bio-enabled nanomaterials, guided-wave systems, micro-fluidics and graphene.

## 1. Introduction

The first observation of enhanced Raman spectra of pyridine on rough silver film was reported in 1974 [1], but the increment of Raman signals was incorrectly attributed to a larger number of molecules on the corrugated surface of the electrode. In 1977, Van Duyne and Albrecht discovered surface-enhanced Raman scattering (SERS) [2,3] as a new phenomenon with extraordinary enhancement of Raman signals from molecules that are in the proximity of metallic nanostructures. Since then, the research interest of SERS has witnessed exponential expansion over the past 38 years, benefitting from a series of discoveries, theories and technological advancements [4,5,6]. In the most recent decade, the explosion of SERS research has majorly been driven by the rapid progress of nanotechnologies [7,8,9] and the incentives for chemical and biomedical applications [10,11,12,13], all of which have greatly fueled the current research endeavors in this topic.

As a highly-sensitive vibrational spectroscopy that allows for the detection of analytes at extremely low concentrations [14,15,16,17], SERS probes the vibrational spectra of various molecules through the enhancement of metallic nanostructures. Consequently, the enhancement factors (EFs) of SERS are crucially dependent on the substrate. In general, there are two important mechanisms underlying SERS [18,19]. The first and also the dominant mechanism towards large EF is due to the electromagnetic field enhancement [20,21], where localized surface plasmons (LSPs) of metallic nanostructure increase the Raman signal intensity. Depending on the structure of the supporting plasmonic material, electromagnetic EF for SERS is theoretically calculated to reach ~10^10^–10^11^ [22]. The other contribution to SERS EFs is due to the chemical enhancement mechanism [23,24], where the charge transfer mechanism between the adsorbed molecule and metal plays a critical role in enhancing and modifying the modes of molecular vibration. Theoretically, chemical EFs up to 10^3^ were calculated using time-dependent density functional theory for para- and meta-substituted pyridine interacting with a silver cluster, but were highly molecular dependent [25,26].

Many years of research have been dedicated to creating and optimizing SERS substrates with large density “hot-spots” in order to provide the largest EFs [8,27,28] based on the primary enhancement mechanism. “Hot-spots” are locations in the vicinity of the plasmonic nanostructures where the local optical field is enhanced tremendously when compared to its surrounding. Any molecule in an SERS-active hot-spot will exhibit an enormous enhancement in its Raman scattering signals. The fundamental metric for SERS activity is the single molecule enhancement factor (SMEF), which quantifies the increase in signal intensity (counts·s^−1^·mW^−1^) per molecule. The SMEF to SERS can be simply expressed as [29]:

(1) $$SMEF\approx M_{Loc}(\omega_{L})M_{Rad}(\omega_{R})\approx \frac{{\left|E_{Loc}(\omega_{L})\right|}^{2}}{{\left|E_{Inc}\right|}^{2}}\frac{{\left|E_{Loc}(\omega_{R})\right|}^{2}}{{\left|E_{Inc}\right|}^{2}}$$

where *M_Loc_* is the local field intensity enhancement; *M_Rad_* is the radiation enhancement factor; and *ω_L_* and *ω_R_* are the resonant angular velocities of the local field *E_loc_*; and radiation field *E_rad_*, respectively. In many cases, the Raman shift is small, and one can make additional approximation that *ω_L_ = ω_R_.* This leads to the famous expression of the SERS enhancement in the |*E*|^4^-approximation as:

(2) SMEF≈|ELoc(ωL)|4|EInc|4

Therefore, enhancing the localized electric field is the most effective method to increase the SERS sensitivity. Typical SMEF of plasmonic materials ranges from 10^4^ to 10^7^, with a few highly-enhancing substrates achieving 10^8^–10^12^ [30,31,32], which is sufficient for single molecule detection.

Initially, SERS substrates were created by depositing metal films or electro-chemically roughening metal electrodes [2]. Then, silver (Ag) and gold (Au) colloids prepared by wet chemical synthesis processes were used [33,34]. In such SERS substrates, light concentration occurs preferentially in the gaps, crevices or sharp features of plasmonic materials with nanoscale features. For instance, metal dimers with gaps less than 5 nm achieved extremely strong SERS signals [35,36]. However, such “hot-spots” (especially <1 nm gaps) with enhanced LSPs are produced only randomly. From the engineering perspective, it is not a controllable technology. In order to improve the reproducibility, there have been a number of innovations in nanofabrication and synthesis of plasmonic nanostructures [37,38]. With the ability to control the size, shape and orientation of plasmonic nanostructures on a surface, researchers have reduced many of the complex variables related to SERS and greatly enhanced the reproducibility of SERS substrates. Such rationally-designed SERS biosensors have gained a tremendous amount of research interest [8,39,40]. In addition to optimizing the conventional SERS substrates using bottom-up chemical synthesis processes and top-down nanofabrication techniques, there has been a new research trend in hybrid SERS substrates by integrating plasmonic nanostructures with other nano-photonic platforms, such as gratings [41], photonic crystals [42], hollow-core waveguides [43], resonant cavities [44] and micro-fluidics [45]. Such hybrid SERS substrates can provide unprecedented sensitivity, high reproducibility and add new functionality into existing SERS substrates.

## 2. Review of Plasmonic Nano-Structures by Bottom-Up Chemical Synthesis Processes

### 2.1. Plasmonic Nanoparticles 

Nobel metal nanoparticles, especially for Au and Ag nanoparticles (NPs), are commonly-used SERS-active substrates. The bottom-up chemical synthesis process of AuNPs and AgNPs with spherical geometry is a simple, rapid and cost-effective strategy. Many of the typical methodologies to prepare NPs were achieved by co-precipitation of soluble metal salt in aqueous solutions followed by relevant reducing agent added [34,46,47,48,49]. The most extensively-employed method for the preparation of plasmonic NPs is Lee and Meisel’s method [34], which is based on sodium citrate reduction of AgNO_3_ under thermal condition and the resultant AgNPs with an average size of ~60 nm. Frens’s and Natan’s methods are the most commonly used to fabricate AuNPs [48,49], in which the AuNPs with different diameters were obtained by adjusting the amount of sodium citrate added. Both of these methods have been mostly employed in various SERS studies. The diameter of plasmonic NPs is crucial for SERS performance, because the local electromagnetic field on the surface of nanoparticles could be harnessed through varying the size of the nanostructures [8,50,51,52].

**Figure 1:**
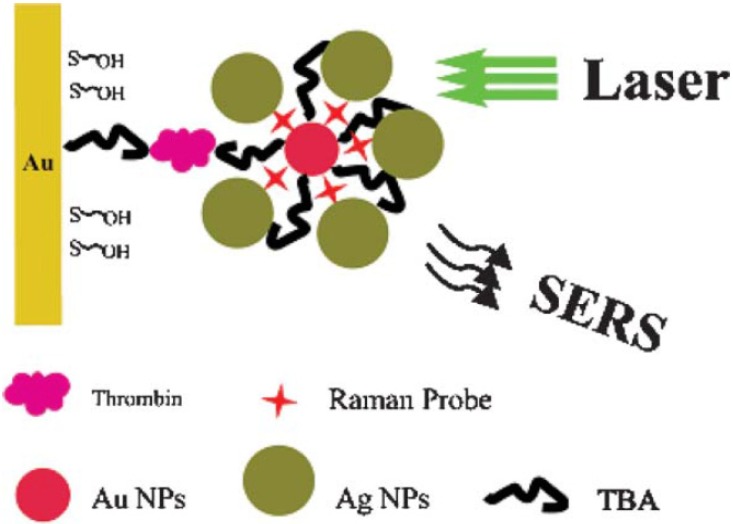
Schematic illustration of the fabrication process of a surface-enhanced Raman scattering (SERS) biosensor for protein recognition. AuNPs with a diameter of around 13 nm labeled with antibody and the Raman probe molecule were used as immuno-gold colloids, which were crosslinked onto the solid chip during the process of immunoassay detection. After the Ag colloids’ deposition, the SERS signal of the Raman probe was obtained [56]. (Reproduced with permission from Wang *et al*., published by Royal Society of Chemistry, 2007.)

It well known that AgNPs exhibit much higher EFs and boost SERS signals that are several times higher than similar AuNPs in the visible wavelength region. Nie’s and Kneipp’s group have obtained Raman spectroscopy of a single molecule on the surface of Ag nanostructures with EF as high as 10^14^ [32,53]. Due to the excellent bio-compatibility, AuNPs SERS sensing has plenty of usages in bioanalysis [54] although suffering moderate sensitivity. To improve the detection limit, immuno-gold colloids associated with AgNP deposition has been demonstrated to achieve a lower limit of detection [55,56]. Wang *et al.* [56] reported the preparation of immuno-gold colloids by labeled AuNPs with 15-meraptamers (TBA) and Raman reporters (Rhodamine 6G), a sensing interface with a sandwich-type system of TBA/a-thrombin/TBA–AuNPs was fabricated as shown in Figure 1. Hot-spots for SERS can be fabricated by deposition of AgNPs on a chip, and a large plasmonic coupling effect is presumably produced at the junction between AuNPs and AgNPs where the Raman reporters reside; high sensitivity with a detection limit as low as 0.5 nM was achieved.

Another important category of plasmonic NPs for SERS is hybrid nanomaterials, because the bimetallic alloy nanoparticles not only provide an enhanced effect for SERS, but also could have new functionalities, such as catalysts and magnetic separation [57,58,59,60]. Sun *et al.* [58] have synthesized binary metallic nanoparticles of Au-Pd in an eco-friendly way based on vitamin C and cacumen platycladi leaf at room temperature, which exhibited an excellent SERS activity and efficient catalytic ability.

### 2.2. Core-Shell Nanoparticles

**Figure 2:**
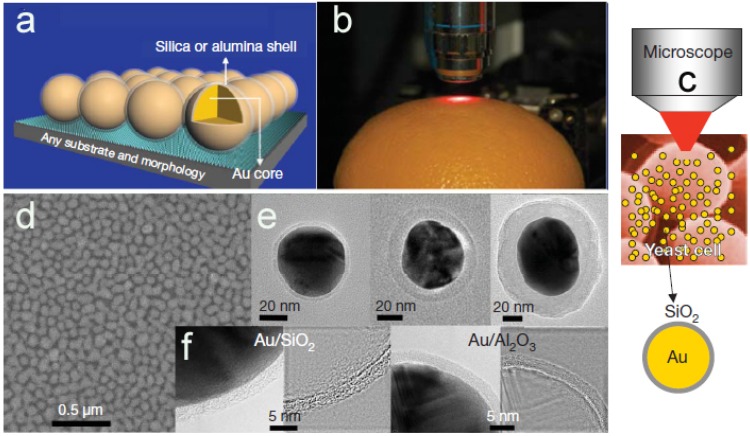
(**a**) The working principles of the shell-isolated nanoparticle-enhanced Raman spectroscopy (SHINERS) mode; (**b**) *in situ* inspection of pesticide residues on fruit by SHINERS; (**c**) *in situ* probing of biological structures by SHINERS; (**d**) SEM image of a monolayer of Au/SiO_2_ nanoparticles on a smooth Au surface; (**e**) HRTEM images of Au/SiO_2_ core-shell nanoparticles with different shell thicknesses; (**f**) HRTEM images of Au/SiO_2_ nanoparticle and Au/Al_2_O_3_ nanoparticle with a continuous and completely packed shell about 2 nm thick [67]. (Reproduced with permission from Li *et al*., published by Nature Publishing Group, 2010.)

The main problems of Au- and Ag-NPs for SERS sensing are the instability under ambient conditions, oxidation in air and aggregation in saline, especially under physiological conditions or in the presence of high-concentration salts [61]. To overcome these drawbacks, Au- and Ag-NPs are usually coated with shells to form the core/shell nanostructures. The shells not only protect Au- and Ag-NPs from aggregation for enhanced stability, but also allow enabling new functionality for the SERS substrates [61,62]. A pioneering work on silica coating was reported by Liz-Marzan *et al.* [63] together with the mechanism of the coating process. Nie’s and Natan’s groups fabricated silica-coated plasmonic cores embedded with Raman probes and named them SERS tags [64,65], in which silane coupling agents were necessary to make nanoparticle surfaces vitreophilic (affinity for silica). Du’s group developed a simple method to prepare silica-encapsulated Ag (Ag@SiO_2_) Raman tags embedded with the Raman reporter molecules, using environmental-friendly solvents (mixed solvents of ethanol and water) without vitreophilic pretreatment [61,66]. The core-shell structure SERS tags exhibited excellent stability and were successfully applied for biomolecule detection. An innovative approach named shell-isolated nanoparticle-enhanced Raman spectroscopy (SHINERS) was exploited recently by Tian and co-workers [67,68], which was based on AuNPs coated with an ultra-thin layer of silica or alumina shell free of Raman reporters, as shown in Figure 2. The ultrathin shell coating on the AuNP is used to protect the plasmonic Au core from contact with the targets and enhanced the stability of SERS substrates. Such shell-isolated plasmonic NPs could be randomly spread as “smart dust” onto the probe surface.

In addition to the aforementioned metal-core/dielectric-shell nanostructures, binary metal core-shell structures are also important for SERS substrates, such as magnetic@AgNPs and Au@Ag nanoparticles [69,70,71]. The magnetic core could be readily used for separation or medical imaging, while the plasmonic Ag shell was used as the SERS active substrate. Au@AgNPs took advantage of the higher SERS activity of silver and the homogeneous superiority of gold.

### 2.3. Plasmonic Nanowires and Nanorods

**Figure 3:**
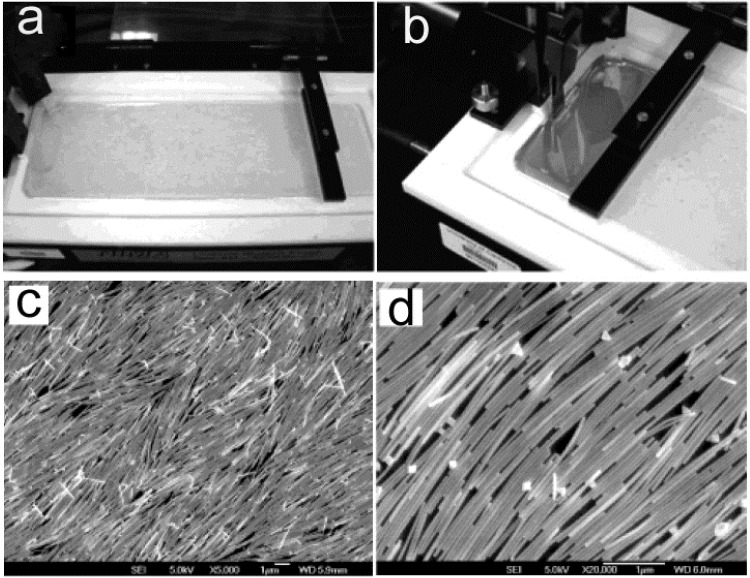
Photographs of Langmuir–Blodgett (LB) nanowire assembly process (**a**,**b**) at different compression stages. SEM images (**c**,**d**) of the Ag nanowire monolayer deposited on a silicon wafer at different magnifications [75]. (Reproduced with permission from Tao *et al*., published by ACS, 2003.)

Metallic nanowires are unique plasmonic nanomaterials, as they support propagating surface Plasmon polaritons (SPPs) [72,73]. Ag nanowires with a smooth surface show minimal plasmon damping during the propagation due to the well-defined crystal structure and, hence, have been employed in the studies of the SPP-based waveguide. The polyol process was the most employed method to prepare Ag nanowires with diameters in the range of 30–60 nm and lengths up to 50 μm [74]. In the context of SERS, Plasmon coupling of individual plasmonic Ag nanowire to each could create high EFs [75,76]. Tao *et al.* [75] have used Langmuir–Blodgett technique to assemble monolayers of aligned Ag nanowires, as shown Figure 3. The Ag nanowires are close-packed and aligned in parallel with each other, and the significant enhancement of the electromagnetic field could stem from the coupling of SPPs among neighboring plasmonic Ag nanowires. The monolayers of nanowires exhibit excellent performance for SERS.

The band of LSPs of metal nanomaterials is primarily dependent on the morphology of the nanomaterials, as well as their intrinsic permittivity. Au nanorods have two plasmonic bands: the transverse band at lower wavelengths with a position coincident with AuNPs that corresponds to electron oscillations perpendicular to the long axis and a longitudinal band in the longer wavelength position that corresponds to electron oscillation along the long axis [77]. The longitudinal plasmon band is sensitive to the aspect ratio of the nanorods, which could be shifted randomly between the visible and the NIR region, as the rod’s aspect ratio changed by simply varying the condition during the chemical synthesis process, and, hence, has attracted considerable attention. El-Sayed’s group has developed lot of work on Au nanorod synthesis [78]. Surfactant cetyltrimethylammonium bromide (CTAB) was used as a micellar template during the synthesis process. In addition, the theoretical per micrometer absorption coefficients of Au nanorods are over an order of magnitude higher than that observed for Au nanoshells. These merits of Au nanorods have enabled its use as SERS substrates in biological applications, including cell imaging, *in vivo* tumor screening and photothermal heating therapy [79,80].

### 2.4. Preassembled Plasmonic Dimers and Trimers

The electromagnetic field in the junction region between coupled plasmonic NP aggregations can be drastically amplified. The nanogap junction of dimers and trimers of metallic NPs could afford extraordinary EF that is high enough for single-molecule detection [22,32,54]. Moreover, the dimer and trimer of plasmonic NPs have new LSP bands at the long-wavelength region, which could be attributed to the aggregation [81,82]. It is critical to achieve a precise control of the size and shape of the aggregated NPs at the nanoscale. Recently, many innovative strategies have been investigated to fabricate dimers and trimers of plasmonic NPs for SERS study. The salt-induced aggregation method is the most commonly used to fabricate nano-aggregates [22,81,82,83]. Chen *et al.* [83] firstly added NaCl to metallic NPs to induce aggregation and then encapsulated the nano-aggregates with polymer. After that, the monomers, dimers and trimers were separated by a differential centrifugation. The authors recorded SERS spectra of the isolated nanostructures, and the respective EFs of these substrates were obtained. The single-stranded DNA, thiol-linkers and organic molecules also have been employed to assemble AuNPs into dimers and trimers [84,85,86,87].

### 2.5. Plasmonic Nanoprisms, Nanocubes, Nanostars and Nanosheets

**Figure 4:**
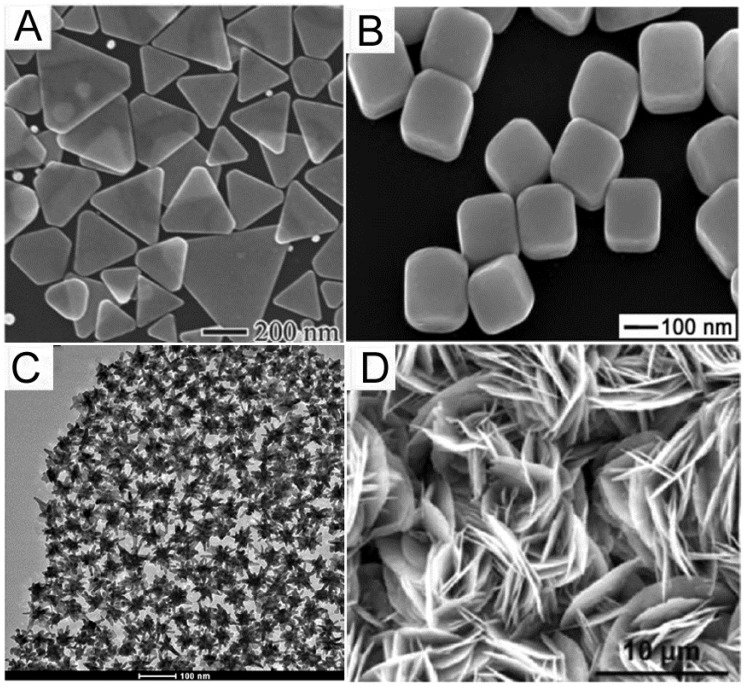
Representative metallic nanomaterials with different geometric morphologies: (**A**) SEM images of Ag nanoprisms [89] (reproduced with permission from Yang *et al*., published by John Wiley and Sons, 2014); (**B**) SEM images of Ag nanocubes [90] (reproduced with permission from Sun *et al*., published by AAAS, 2002); (C) TEM images of Au nanostars [94] (reproduced with permission from Yuan *et al*., published by ACS, 2012); (**D**) SEM images of Ag nanosheets [96] (reproduced with permission from Yan *et al*., published by ACS, 2012).

Other metallic colloids with unique geometric morphologies, such as nanoprisms [88,89], nanocubes [90,91], nanostars [92,93] and nanosheets [94,95], that were investigated as SERS substrates have also inspired great interest, because the sharp edges or branches of metallic nanostructures could concentrate charge and cause greater electric fields than those of spherical nanoparticles. Yang *et al.* [89] synthesized Ag nanoprisms (Figure 4A) by an etching process, in which the AgNO_3_ were firstly reacted with poly(vinyl pyrrolidone) (PVP) in an aqueous solution under a hydrothermal condition to form Ag nanoplates; the sharp features on nanoprisms with wavy edges are very active for SERS. Xia and co-workers developed a modified polyol process to prepare Ag nanocubes with sharp and truncated edges, as shown in Figure 4B, in which ethylene glycol functions as the solvent and reducing agent [90]; they also observed that the intensity of SERS signals of probe molecules on nanocubes with sharp edges were dependent on the polarization of the excitation laser [91]. This angular dependence was less significant when the nanocubes had a nearly spherical profile. Nanostars (as shown in Figure 4C) are a kind of multi-branched nanomaterial [94], and large SERS EFs could be obtained on the surface of nanostars due to the ‘sharp tip effect’ of branches [92]. Additionally, the plasmon hybridization between individual tips and cores gives rise to a further increase in the local electric field. Nanosheet assemblies with well-defined and reproducible structures are shown in Figure 4D [96], which could be fabricated by growing metallic materials on the polyaniline surfaces, and the size and density of the nanosheets can be manipulated easily during the process of chemical synthesis. As SERS substrates, nanosheet assemblies are reproducible, highly sensitive and facile for fabrication.

## 3. Review of Rationally Designed SERS Substrates 

The progress of SERS sensing using conventional bottom-up chemical synthesis has been hampered by the inability to fabricate substrates with the high sensitivity, tunability, robustness and reproducibility necessary for truly practical and successful SERS-based systems. These limitations are mostly due to the lack of control over the nanoscale features of the plasmonic substrates that are responsible for the enhancement. With the advent of advanced nanotechnologies, new approaches have been developed to overcome these challenges and to produce substrates with higher sensitivity, stability and reproducibility. This section will review recent progress in the rationally-designed SERS substrates using both fine-tuned bottom-up and top-down fabrication techniques, with an emphasis on the influence of nanotechnology.

### 3.1. Nanosphere Lithography

**Figure 5:**
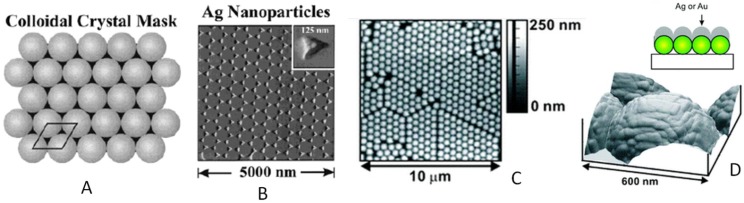
Schematic illustration of a colloidal crystal mask (**A**) and representative AFM image (**B**) of Ag nano-triangular structures. Ambient contact mode atomic force microscope image of 200-nm Ag over 542-nm diameter polystyrene spheres: (**C**) array of spheres (10 μm × 10 μm) and (**D**) image (600 nm × 600 nm) of one sphere showing substructure roughness with schematic illustration [97,101]. (Reproduced with permission from Van Duyne *et al*., published by ACS 2001, 2002.)

Nano-triangle structures have sharp edges at their vertex that can concentrate optical fields within narrow gaps and, hence, can be utilized for SERS sensing. The most effective method to produce nano-triangle patterns was invented by Van Dyune and co-workers [97,98,99] using nanosphere lithography (NSL), which is an inexpensive, versatile and inherently parallel lithography method. First, uniformly-sized nanospheres are self-assembled on a glass substrate to form a 2D colloidal crystal deposition mask (Figure 5A). Second, a thin layer of metal film (e.g., Au or Ag) is deposited either by thermal evaporation or electron beam deposition, or pulsed laser deposition through the nanosphere mask. Next, the nanosphere mask is removed by sonication of the sample in a solvent, thereby leaving behind the nano-triangle metal patterns deposited in the interstitials of the nanosphere mask (Figure 5B). Interestingly, a complementary plasmonic nanostructure called metal film over nanosphere (MFON) [100,101] has also been generated by directly depositing metal film on the nanosphere array (Figure 5C,D). The most critical control of NSL is the inter-particle spacing, which is of paramount importance to electromagnetic enhancement mechanisms. In order to prepare a substrate with the maximum amount of hot-spots with minimized gap width, Halas *et al.* [102] functionalized 50-nm AuNPs with cetyltrimethylammonium bromide (CTAB), re-dispersed the colloids in water and finally drop cast the suspension to create a monolayer of particles with an average inter-particle distance of ~8 nm, as a self-assembled CTAB bilayer on the AuNPs generates a net positive charge on each particle, which leads to electrostatic repulsion that prevents random aggregation. This gap provides the strong near-field enhancement.

### 3.2. On-Wire Lithography

**Figure 6:**
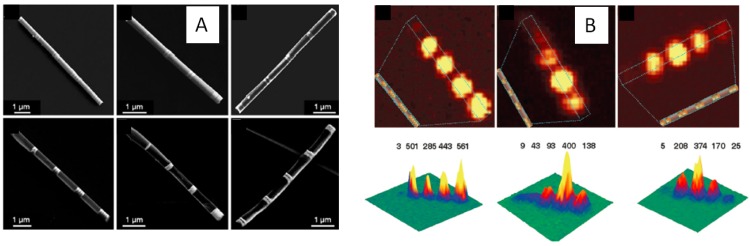
(**A**) SEM images of on-wire lithography (OWL)-generated nanodisk arrays with various geometries; (**B**) Confocal Raman microscopy images of gapped nanowire structures functionalized with methylene blue (MB) [104]. (Reproduced with permission from Qin *et al*., published by National Academy of Sciences, USA, 2006.)

On-wire lithography (OWL) has emerged as a powerful method of synthesizing a segmented structure and subsequently introducing architectural changes through post-chemical treatment [103,104,105,106,107]. OWL is based on the idea that one can make segmented nanowires consisting of at least two types of materials, one that is susceptible and one that is resistant to wet chemical etching. Mirkin’s group grew multi-segmented (Au-Ni) nanowires by electroplating inside an alumina template and successive etching of the template. After selective chemical etching of the sacrificial segments (Ni), various nanostructures with gaps as small as 2 nm and disks as thin as 20 nm can be created. Using this method, the authors were able to synthesize arrays of dimers, trimers, tetramers and pentamers for SERS sensing with varying geometries synthesized on one array, as shown in Figure 6A. These tunable arrays allow for internal controls and direct comparison of the relative Raman intensity of each structure with a confocal Raman microscope, as shown in Figure 6B. Furthermore, of note is that these arrays were dispersible, allowing for colloidal functionalization techniques. The authors found that 120 nm-thick dimers with a 30-nm gap spacing produced the optimal response (over 100× the intensity of a single disk) for 633-nm excitation lasers. These experimental results were correlated with theoretical models using the discrete-dipole approximation (DDA). The DDA approach predicted that the maximum EF generated by 120 nm-thick disks separated by 10 nm provided the optimal response (Figure 2B). The breakthrough of OWL makes it possible to control the gap size between metal nanoparticles in a practical way and has demonstrated remarkable sensitivity to chemical molecules, such as methylene blue (MB), p-mercaptoaniline and Cy-3-labeled DNA. However, the density of such nanogaps is limited, for only a few nanogaps can be created along a nanowire, which requires a relatively high concentration of molecules to increase the detection probability.

### 3.3. Plasmonic Nano-Capsules and Electric Tweezers

**Figure 7:**
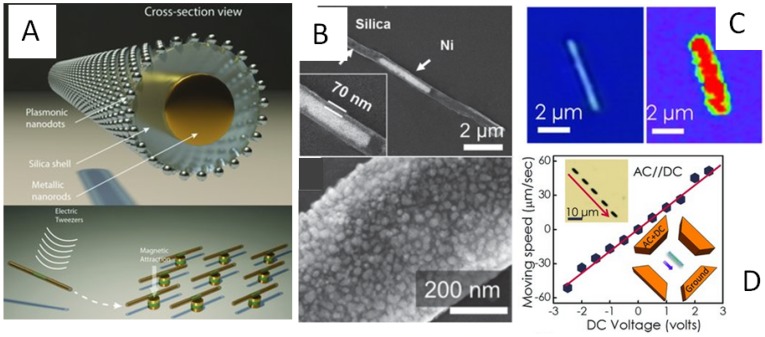
(**A**) (Top) Structure of a tri-layer nanocapsule; (bottom) with the electric tweezers, nanocapsules can be transported and assembled onto a pre-patterned array of nanomagnets by utilizing the magnetic attraction force between the Ni segments in the nanocapsules and the magnetic layers inside the nanomagnets [108] (reproduced with permission from Xu *et al*., published by John Wiley and Sons, 2012); (**B**) SEM images of silica nanotubes embedded with Ni nanomagnets and a close view of the AgNPs on the nanotube surface [109] (reproduced with permission from Xu *et al*., published by John Wiley and Sons, 2013); (**C**) Raman mapping profile of 1 μM Rhodamine 6G dispersed on a tri-layer nanocapsule shows uniform SERS intensity on the entire surface of the nanocapsules. (1655 cm^−1^, scan step 250 nm, integration time 0.5 s) [109] (reproduced with permission from Xu *et al*., published by John Wiley and Sons, 2013); (**D**) Electric tweezers using AC and DC configurations on quadruple electrodes for the manipulation of nanocapsules [108] (reproduced with permission from Xu *et al*., published by John Wiley and Sons, 2012).

Conventional SERS substrates are immobile, while mobile SERS tags cannot provide enough hot-spot density. In order to create a large number of hot-spots and simultaneously achieve flexible assembly capability at desirable positions for location predicable sensing, Fan *et al.* [108,109] invented plasmonic nano-capsules. A plasmonic nanocapsule consists of a tri-layer structure with a three-segment Ag/Ni/Ag nano-rod as the core, a thin layer of silica as the capsulating layer and uniformly-distributed AgNPs on silica as the hotspot layer, as illustrated in the upper figure of Figure 7A. The outermost layer made of dense AgNPs with optimized sizes and junctions provides a large number of hotspots (about 1200 μm^2^) for ultrasensitive SERS detection, as shown in Figure 7B. The nanocapsules exhibited an absorption peak at 450 nm due to the collective plasmonic resonance of assembled AgNPs. Using a 532-nm excitation laser, the nanocapsules detected Raman spectra of 1,2-bi-(4-pyridyl) ethylene (BPE) with a concentration as low as 10^−12^ M (1 pM). The mapping results of the Raman signal in Figure 7C also clearly confirmed the high-density hot-spots throughout the nanocapsules. More interestingly, the plasmonic nanocapsules can be transported and assembled into ordered arrays using an innovative manipulation technique called “electric tweezers” [110], as shown in Figure 7D. A prototype of a 3 × 3 nanocapsule sensor array has demonstrated the ability to successfully detect various biomolecules, as shown in Figure 7D and the lower figure of Figure 7A. Leveraging the bi-functionality, a plasmonic nanocapsule is magnetically maneuvered to a single living mammalian cell, and its membrane composition is analyzed via SERS spectroscopy [111].

### 3.4. Plasmonic Nano-Antennas by E-Beam Lithography

Optical nano-antennas are devices that convert freely-propagating optical radiation into localized energy at nanometer scales (hot-spots), and *vice versa* [111,112,113,114]. The huge electromagnetic field enhancement at the hot-spots enables a dramatic increase of the vibrational signal of molecules located therein. In the few past years, SERS EF up to 10^8^ has been reported [115], allowing one to carry out vibrational analysis down to the single-molecule level. Moerner *et al.* [116,117] fabricated SERS substrates containing Au “bowtie” nanostructures. Each ‘‘bowtie’’ consisted of two electron-beam lithographically (EBL)-generated triangles approximately 100 nm on a side, 18 nm thick and with a sub 20-nm junction formed at the junctions of two vertices, one from each triangle. The “bowtie” antennas were coated with pMA, and the enhancement mechanism was studied. As the structures were rationally designed and the dimensions were stable and highly reproducible, the electromagnetic (EM) contribution to the SERS response was deterministic. Accordingly, variations in chemical enhancement (CE) would be responsible for observed variations in the EF if any existed. In their study, they found that Raman modes corresponding to the aromatic character of the pMA, where the aromatic ring can switch from standing vertically away from the surface to lying flat on the Au surface, exhibit wide variations in EF; modes not greatly affected by these orientation changes (e.g., C–S stretches in pMA) do not exhibit high variability in their Raman signature. This work is one illustration of how a rationally-designed substrate could be engineered with high reproducibility to probe the fundamental mechanisms of SERS. Another popular plasmonic nano-antenna structure is the Yagi-Uda antenna [118,119], and the application for SERS has been reviewed [120]. Pucci *et al.* [121] fabricated gold nano-antennas by EBL on calcium difluoride (1–2 μm long, 60 nm wide, 60 nm high) that exhibit a transverse plasmonic resonance in the visible (640 nm) for SERS and a particularly strong longitudinal dipolar resonance in the infrared (tunable in the 1280–3100 cm^−1^) for surface-enhanced infrared absorption (SEIRA), which is another important plasmonic-enhanced vibrational spectroscopy technology.

### 3.5. Nanogaps Created by Non-Traditional Techniques

**Figure 8:**
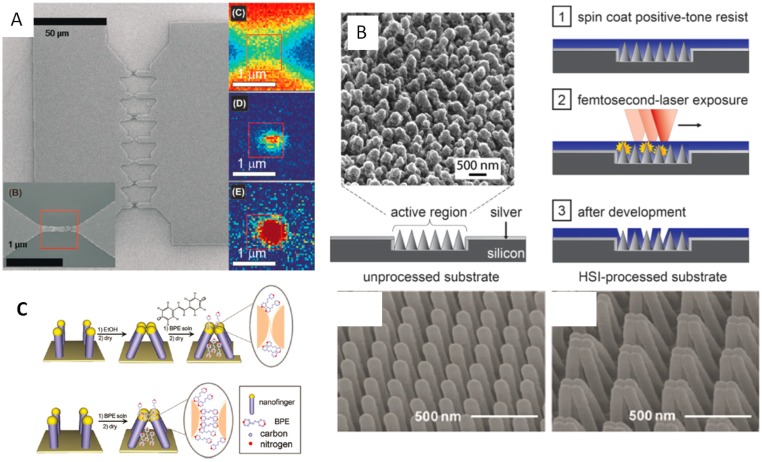
(**A**) Electro-migrated gaps from [122] (reproduced with permission from Ward *et al*., published by ACS, 2007). (**B**) Helium ion microscope image (45°) and schematic diagram of multiphoton-lithography SERS substrate (left). Hot-spot isolation (HSI) process (right): (1) diluted positive-tone photoresist is spin-coated onto a SERS substrate to cover the surface; (2) a femtosecond laser pulse train is scanned over the surface, selectively exposing the photoresist covering electromagnetic hot-spots; (3) the photoresist is developed, and the SERS hot-spots are uncovered [123] (reproduced with permission from Diebold *et al*., published by ACS 2009). (**C**) (Left) Schematic illustrations of the preparation of preclosed and 1,2-bi-(4-pyridyl) ethylene (BPE)-trapped gold fingers. The inset shows the magnified view of finger tips. (Right) SEM images of as-fabricated and closed fingers [127] (reproduced with permission from Kim *et al*., published by ACS, 2011).

The major difficulty of fabricating plasmonic nano-antennas by EBL is the extremely small feature size, particularly the narrow gaps between the antennas as the hot-spots, which were limited by the proximity effect. To achieve even smaller metallic nanogaps, EBL was combined with electrochemical methods for the fine-tuning of gap dimensions. Halas and co-workers started the fabrication with EBL to pattern a series of “bowtie” structures that consist of two larger (~50 μm × 150 μm) pads connected by thin, tapered bridges with a 80–100-nm width [122]. By applying electric current though these thin junctions, the momentum from current-carrying electrons was transferred to the Au lattices, impacting atomic positions, eventually leading to a break in the thin Au section (Figure 8A). The resulting atomic migration-created gaps have widths ranging from <1 nm to a few nm, which can be manually or automatically controlled by monitoring the resistance of the junction. SERS spectra measured at the nanogaps showed high EFs along with blinking and spectral diffusion, indicating that the sensitivity was approaching the single molecule level. This method of electro-migration on lithographically-formed features can generate highly reproducible SERS hot-spots, with EFs easily reaching the 10^8^ range. Recently, Diebold *et al.* [123] reported a method to screen hot-spots by a femtosecond pulsed laser beam. In this study, a nanostructured Ag-covered silicon surface is covered by a positive photoresist, and a femtosecond laser pulse train is scanned over the surface, selectively exposing the photoresist covering electromagnetic hot-spots. The authors conclude that only the hot-spots are developed, based on the assumption that the multiphoton-induced luminescence is greatest in the hot-spot. The schematic and the resulting substrate are shown in Figure 8B. SERS substrates directly fabricated by a femtosecond laser, which has rippled nanostructures, were also reported [124]. The formation of electromagnetic hot-spots can also be achieved by mechanical assembly via convective flow upon solvent evaporation. Li *et al.* [125,126,127] demonstrated a structure based on gold-coated nanoscale polymer fingers made by a nano-imprinting technique. The so-called “nano-fingers” were immersed in an ethanol solution containing analyte molecules. Nano-finger assembly and aggregation is induced by capillary forces upon solvent evaporation. The assembled nano-fingers are depicted in Figure 8C. After drying, the analyte molecules are trapped in the molecule self-limiting gaps between fingertips, which ensures ultimate SERS enhancement for sensitive molecule detection. The closed finger samples displayed an increased signal by 10×–30× over one without solvent exposure. The authors estimate an EF of 2 × 10^10^. Besides the high EFs, another major benefit of this technique is the resulting long-range order.

### 3.6. Plasmonic Gratings and Other Periodic Metallic Structures

Grating-type substrates with nanometer dimensions offer the possibility of enhancing the electromagnetic field close to surfaces by SPP excitation. Although not as popular as other rationally-designed plasmonic nanostructures due to the relatively low EFs, the well-defined topology of plasmonic gratings was used for quantitative comparison of theory and experiment [128]. Optimization of the grating depth has been investigated to maximize the SERS EF using rigorous coupled wave analysis (RCWA) methods [129,130]. Experimental results on a silver grating of variable groove depth coated by a thin film of copper phthalocyanine showed an increase of the Raman signals of 10^4^ as compared to the flat case (copper phthalocyanine on a flat silver surface) [131]. To overcome the polarization dependence of 1D grating, Grande *et al.* [132] reported the results of 2D gold nano-patch arrays on a silicon substrate and found a dramatic improvement in the Raman signal when the laser wavelength matches the SPPs of the periodic nanostructures. SERS mapping estimated the SERS EFs ~2 × 10^5^. Weiss *et al.* [133,134] demonstrated large area two-dimensional arrays of patterned nano-porous gold as easy-to-fabricate, cost-effective and stable SERS templates. Using a simple one-step direct imprinting process, subwavelength nano-porous gold (NPG) gratings are defined by densifying appropriate regions of a NPG film. Both the densified NPG and the two-dimensional grating pattern are shown to contribute to the SERS enhancement. The resulting substrates exhibit uniform SERS enhancement factors of at least 10^7^ for a monolayer of adsorbed benzenethiol molecules.

## 4. Review of Hybrid SERS Substrates on Other Nanophotonic Platforms

### 4.1. SERS Substrates on Photonic Crystals and Resonant Gratings

**Figure 9:**
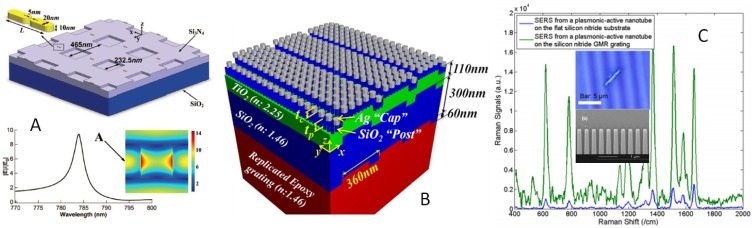
(**A**) (Top) The geometry of the dielectric grating structure with silver nanorod optical antennas on 2D silicon nitride grating surface. (Bottom right) Field map |E|/|E_0_| at the surface of the grating at resonance. (Bottom left) |E|/|E_0_| *versus* wavelength calculated by rigorous coupled wave analysis (RCWA) at Point A as marked on the inset to the right. |E_0_| is the magnitude of the incident electric field [138] (reproduced with permission from Li *et al*., published by American Institute of Physics, 2009). (**B**) Schematic diagram of PC-SERS substrate of SiO_2_-Ag “post-cap” nanostructure with a thickness of the SiO_2_ layer of 75 nm and a thickness of the Ag layer of 20 nm by the oblique angle deposition method [139] (reproduced with permission from Kim *et al*., published by Optical Society of America, 2010). (**C**) Measured 1 µM R6G Raman spectrum from the plasmonic nanotube on the flat Si_3_N_4_ substrate and from the plasmonic nanotube on the GMR grating. The inset figures show the SEM image of the grating and the optical image of the plasmonic nanotube on top of the grating [41] (reproduced with permission from Xu *et al*., published by American Institute of Physics, 2012).

Directly using plasmonic gratings as the SERS substrates can only provide limited EFs. However, integrating plasmonic NPs with dielectric gratings or photonic crystals can achieve extremely high SERS EFs. Guided-mode resonance (GMR) is a phenomenon wherein the guided modes of an optical waveguide can be excited and simultaneously extracted by the introduction of a phase-matching element. Such guided modes are also called “leaky modes”, as they do not remain guided, and have been observed in one- and two-dimensional photonic crystal slabs [135] and dielectric gratings [136]. GMRs in dielectric gratings have also been discovered to enhance the LSPs of plasmonic NPs, which will further increase the SERS signals [137,138]. The working mechanism relies on resonant modes when properly excited, which can serve as a universal field-enhancement substrate, as illustrated in Figure 9A. Cunningham *et al.* [139] coupled a tightly-packed layer of discrete metal nanoparticles to the GMR of a photonic crystal surface. Because metal nanoparticles introduce absorption that quenches the photonic crystal resonance, a balance was achieved between locating the metal nanoparticles too close to the surface while still positioning them within the enhanced evanescent field to maximize coupling to surface plasmons. The photonic crystal-SERS substrate was comprised of a replica molded photonic crystal slab as the dielectric optical resonator, a SiO_2_ “post” layer thickness of 50 nm and a Ag “cap” height of ~20 nm, as shown in Figure 9B. As a simpler approach, Wang and Fan *et al.* [41] demonstrated a photonic crystal-SERS substrate by placing plasmonic active SiO_2_ nanotubes on Si_3_N_4_ gratings. As the density of hot-spots is high while the absolute number of AgNPs is relatively low, the quenching effect is low, and the substrate showed a constant enhancement factor of 8×~10× in addition to the existing SERS effect of SiO_2_ plasmonic nanotubes [41], as depicted in Figure 9C. One challenge for this approach is how to fine-tune the GMR frequency of the dielectric grating to match the excitation wavelength. Wang *et al*. [140] showed that indium-tin oxide (ITO) gratings can achieve excellent tolerance to fabrication deviations due to the large refractive index contrast of the ITO grating. Quantitative experimental results of 5,5’-dithiobis(2-nitrobenzoic acid) (DTNB) demonstrate the best enhancement factor of ~14× on ITO gratings when compared with AgNPs on a flat ITO film, and the limit of detection (LOD) of DTNB is as low as 10 pM.

### 4.2. SERS Substrates Using Bio-Enabled Materials

**Figure 10:**
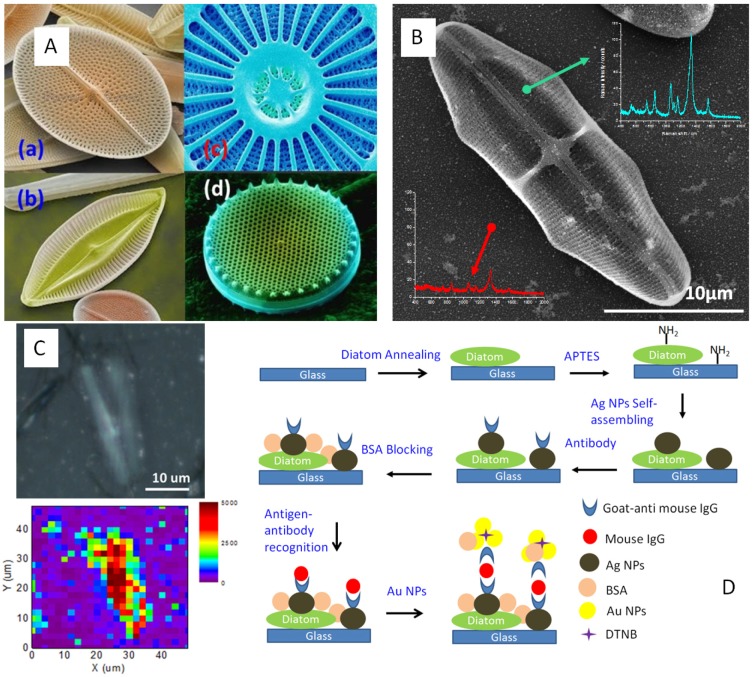
(**A**) SEM images of various diatom species with different frustules [141] (reproduced with permission). (**B**) SEM image of the hybrid diatom-plasmonic SERS substrates [151] (reproduced with permission from Yang *et al*., published by John Wiley and Sons, 2014). (**C**) The 1 μM R6G Raman mapping results from the hybrid diatom-plasmonic SERS substrate [150] (reproduced with permission from Ren *et al*., published by IEEE, 2014). (**D**) Schematic diagram of the diatom-plasmonic SERS immunoassay using a standard antigen-antibody model.

Rationally-designed SERS substrates require expensive top-down nanofabrication methods, such as EBL and reactive ion etching (RIE). On the other hand, nature provides an inspirational source of nanophotonic structures. Many photosynthetic marine micro-organisms efficiently capture sunlight for photosynthesis by imbedding inorganic periodic photonic structures, which are very similar to artificial photonic crystals, into their cell walls. Diatoms take up water-soluble silicic acid from the environment, which is then precipitated into amorphous silica within an intracellular nano-bioreactor to form the photonic crystal-like frustules at ambient temperatures and pressures. Different species of diatoms based on biosilica and coccolithophores based on calcium carbonate with versatile photonic crystal structures have been reported (Figure 10A (a–d)) [141]. The potential applications of diatoms in solar cells [142], batteries [143], drug delivery [144], electro-luminescence [145], photo-luminescence (PL) [146], nanofabrication templates [147] and selective membranes [148] have been explored by many research groups. Wang *et al.* [149] numerically simulated the field enhancement of diatom frustules to plasmonic NPs and obtained similar field enhancement as rationally-designed dielectric gratings. The hybrid diatom-plasmonic SERS substrates were obtained by self-assembling AgNPs onto the diatom frustules (Figure 10B), which can be obtained by microbiological incubation as detailed in [149]. From the optical transmission experiment, they observed that bare diatom frustule shows a broadband resonance in the visible wavelength from 400–700 nm, while the NPs-on-glass only have two low-Q resonances (~100-nm bandwidth) centered at ~480-nm and ~630-nm wavelengths, which are due to NP clusters and nanorods, respectively. For the NPs-on-diatom structure, significantly stronger optical extinction is observed, and high-Q resonant peaks (~20-nm bandwidth) appear due to hybrid GMR-LSP modes. These experimental results clearly confirm the existence of GMR-LSP coupling. SERS spectra of 1 μM R6G were also measured on the hybrid diatom-plasmonic SERS substrates with the best additional SERS EF of 12× [150]. To confirm the overall SERS enhancement, SERS signals of R6G at 1368 cm^−1^ were mapped in Figure 10C with the optical image of the scanned area included in the inset figure. The strong correlation of the enhancement factors with respect to the shape of the diatom frustule clearly proves the contribution of the bio-enabled nanomaterial. In addition, Wang *et al.* [151] demonstrated immuno-assay SERS sensors using the hybrid diatom-plasmonic substrates. The SERS-based sandwich immunoassay (protocol shown in Figure 10D) is functionalized with goat anti-mouse IgG. The selectivity to detect specific antigens was tested by challenging the immuno-assay sensors with complementary antigen (mouse IgG) and non-complementary antigen (human IgG), respectively. Quantitative comparison of the detection limit shows that the detection limit on glass is 1 μg/L, while the hybrid diatom-plasmonic SERS substrates improve the detection limit to 10 ng/L, which is ~100× better, as shown in Figure 10D.

### 4.3. SERS Sensing Using Optical Fibers

**Figure 11:**
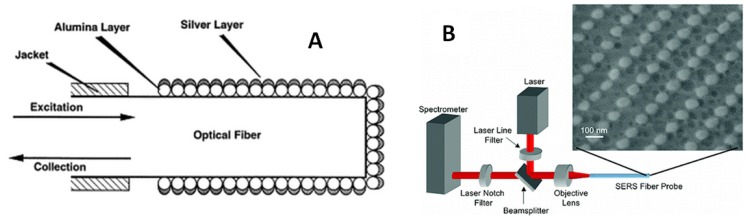
(**A**) Fiber-optic SERS sensors through the incorporation of alumina nanoparticles and silver coatings onto the single mode fiber probe tip [154] (reproduced with permission from Stokes *et al*., published by Elsevier, 2000). (**B**) Schematic depiction of the configuration used to characterize the SERS fiber optic probe and a scanning electron micrograph of an array of gold optical antennas on the facet of a fiber [155]. (Reproduced with permission from Smythe *et al*., published by ACS, 2009).

SERS signals are usually measured through conventional free-space optics using optical microscopes. Although ultra-sensitive due to the large numerical aperture of the objective lens that can focus light into a small spot and a large acceptance angle to collect the Raman signals, it is not convenient to use free-space Raman microscopes for *in situ* or remote sensing. The SERS sensing technique based on guided-wave systems, such as optical fibers [152,153,154,155,156], for irradiating and collecting SERS signals from the SERS substrate can provide better accessibility into complex environment, which will enable *in situ* and *in vivo* sensing capabilities. For example, Vo-Donh *et al.* [154] developed an integrated fiber-optic SERS sensor through the incorporation of alumina nanoparticles and silver coatings onto the single mode fiber probe tip, as shown in Figure 11A. Depending on the tip size, the fiber-optic SERS sensor allows *in situ* measurements at microscale environments. Two detection schemes can be used: a “dip-and-dry” mode of detection and an *in situ* sensing mode were demonstrated for several compounds. A quantitative calibration curve for cresyl fast violet (CFV) in groundwater was established, and a limit of optical detection of approximately 50 ppb was determined. Capasso *et al.* [155] used EBL to fabricate optical antennas on a silicon wafer, which are then transferred onto the fiber facet (Figure 11B). They claimed that they achieved the ability to control the size and spacing of the antennas, which enabled the EF of the transferred array to be estimated from 2.6 × 10^5^ to 5.1 × 10^5^. Recently, several other SERS sensing techniques using liquid-core fibers [157], D-shaped fibers [158] and photonic crystal fibers [43] have also been demonstrated. 

### 4.4. SERS Sensing in Micro-Fluidics

In order to enable on-chip sensing capabilities, the combination of SERS and microfluidic devices is increasingly of particular interest in research, especially in the field of biomedical analytics. The implementation of microfluidic systems in SERS analytics provides the benefit of a highly-defined environment in which the measurement takes place and allows the easy handling of small sample volumes. The integration of functionalities for mixing procedures, sample preparation steps or for the SERS detection itself benefits from a higher reproducibility of the measurements. Schemit *et al.* investigated liquid-core anti-resonant reflecting optical waveguides [159,160] (ARROWs), which provided a high level of integration with other sensor systems. The ARROW was fabricated based on conventional silicon processes and etching of sacrificial core layers. The hollow waveguides with volumes covering the pico- to nano-liter range can confine mode propagation, tail wavelength selectivity and, meanwhile, serve as the microfluidic channels for plasmonic NPs and analyte solution. Other micro-fluidic structures, such as loops [161,162], zigzag-shaped channels [163] or micro-pillars [164], were also introduced to achieve a high mixing efficiency for SERS sensing.

### 4.5. SERS Substrates on Graphene

Graphene is a single sheet of carbon atoms in a 2D honeycomb crystal structure that has exhibited amazing Raman scattering properties, which are related to its unique structure of electrons and phonons. Shen *et al.* [165] deposited Au films on single-layer graphene as SERS substrates for the characterization of R6G molecules. They reported significantly suppressed photoluminescence background by attributing the quenched PL signals to the strong resonance energy transfer from Au to single-layer graphene (SLG). They suggested that the combination of Au films and SLG can be used in the characterization of low concentration molecules with relatively weak Raman signals. Very recently, Zhang *et al*. [166] thoroughly reviewed the multi-roles played by graphene in SERS sensing, including as a Raman probe, as a substrate, as an additive and as a building block for a flat surface.

## 5. Conclusions

In summary, SERS is a highly-sensitive technique that allows for the detection of molecules in very low concentrations and provides rich structural information. As a versatile technique after more than 38 years of research, SERS can not only address fundamental scientific questions, but also can resolve engineering challenges in many applications. However, in all cases, the plasmonic substrate is the most critical component in this field. In this review, we first discussed basic plasmonic materials, such as metallic nanoparticles and nano-rods prepared by conventional bottom-up chemical synthesis processes. Then, we reviewed rationally-designed plasmonic nano-structures created by top-down approaches or fine-controlled synthesis with high-density hot-spots to provide large SERS EFs. Finally, we discussed the research progress of hybrid SERS substrates through the integration of plasmonic nano-structures with other photonic platforms, such as photonic crystals, bio-enabled nanomaterials, optical fibers, micro-fluidics and graphene. We must point out that there has been a very active search for new and better plasmonic materials in recent years [167,168]. It has been postulated that metal oxides, noble-transition alloys, alkali-noble inter-metallic compounds and semi-conductors can exhibit plasmonic properties. Such materials will possibly be employed as new SERS substrates and be utilized for specific applications. Therefore, the searching for better SERS substrates will continue in the future.

## References

[1] Fleischmann M., Hendra P.J., McQuillan A. (1974). Raman Spectra of Pyridine Adsorbed at a Silver Electrode. Chem. Phys. Lett..

[2] Jeanmaire D.L., van Duyne R.P. (1977). Surface raman spectroelectrochemistry: Part I. Heterocyclic, aromatic, and aliphatic amines adsorbed on the anodized silver electrode. J. Electroanal. Chem. Interfacial Electrochem..

[3] Albrecht M.G., Creighton J.A. (1977). Anomalously Intense Raman Spectra of Pyridine at a Silver Electrode. J. Am. Chem. Soc..

[4] Tian Z. (2005). Surface-Enhanced Raman Spectroscopy: Advancements and Applications. J. Raman Spectrosc..

[5] Qian X., Nie S. (2008). Single-Molecule and Single-Nanoparticle SERS: From Fundamental Mechanisms to Biomedical Applications. Chem. Soc. Rev..

[6] Cialla D., März A., Böhme R., Theil F., Weber K., Schmitt M., Popp J. (2012). Surface-Enhanced Raman Spectroscopy (SERS): Progress and Trends. Anal. Bioanal. Chem..

[7] Stewart M.E., Anderton C.R., Thompson L.B., Maria J., Gray S.K., Rogers J.A., Nuzzo R.G. (2008). Nanostructured Plasmonic Sensors. Chem. Rev..

[8] Banholzer M.J., Millstone J.E., Qin L., Mirkin C.A. (2008). Rationally Designed Nanostructures for Surface-Enhanced Raman Spectroscopy. Chem. Soc. Rev..

[9] Sharma B., Fernanda Cardinal M., Kleinman S.L., Greeneltch N.G., Frontiera R.R., Blaber M.G., Schatz G.C., Van Duyne R.P. (2013). High-Performance SERS Substrates: Advances and Challenges. MRS Bull.

[10] Bantz K.C., Meyer A.F., Wittenberg N.J., Im H., Kurtuluş Ö., Lee S.H., Lindquist N.C., Oh S., Haynes C.L. (2011). Recent Progress in SERS Biosensing. Phys. Chem. Chem. Phys..

[11] Rae S.I., Khan I. (2010). Surface Enhanced Raman Spectroscopy (SERS) Sensors for Gas Analysis. Analyst.

[12] Shafer-Peltier K.E., Haynes C.L., Glucksberg M.R., Van Duyne R.P. (2003). Toward a Glucose Biosensor Based on Surface-Enhanced Raman Scattering. J. Am. Chem. Soc..

[13] Anker J.N., Hall W.P., Lyandres O., Shah N.C., Zhao J., Van Duyne R.P. (2008). Biosensing with Plasmonic Nanosensors. Nat. Mater..

[14] Siegel A.C., Phillips S.T., Dickey M.D., Lu N., Suo Z., Whitesides G.M. (2010). Foldable Printed Circuit Boards on Paper Substrates. Adv. Funct. Mater..

[15] Kleinman S.L., Ringe E., Valley N., Wustholz K.L., Phillips E., Scheidt K.A., Schatz G.C., Van Duyne R.P. (2011). Single-Molecule Surface-Enhanced Raman Spectroscopy of Crystal Violet Isotopologues: Theory and Experiment. J. Am. Chem. Soc..

[16] Le Ru E., Meyer M., Etchegoin P. (2006). Proof of Single-Molecule Sensitivity in Surface Enhanced Raman Scattering (SERS) by Means of a Two-Analyte Technique. J. Phys. Chem. B.

[17] Pieczonka N., Moula G., Aroca R. (2009). SERRS for Single-Molecule Detection of Dye-Labeled Phospholipids in Langmuir−Blodgett Monolayers. Langmuir.

[18] Moskovits M. (1985). Surface-Enhanced Spectroscopy. Rev. Modern Phys..

[19] Schatz G.C. (1984). Theoretical Studies of Surface Enhanced Raman Scattering. Acc. Chem. Res..

[20] Moskovits M. (2005). Surface-enhanced Raman Spectroscopy: A Brief Retrospective. J. Raman Spectrosc..

[21] Otto A., Mrozek I., Grabhorn H., Akemann W. (1992). Surface-Enhanced Raman Scattering. J. Phys. Condens. Matter.

[22] Camden J.P., Dieringer J.A., Wang Y., Masiello D.J., Marks L.D., Schatz G.C., Van Duyne R.P. (2008). Probing the Structure of Single-Molecule Surface-Enhanced Raman Scattering Hot Spots. J. Am. Chem. Soc..

[23] Campion A., Ivanecky J., Child C., Foster M. (1995). On the Mechanism of Chemical Enhancement in Surface-Enhanced Raman Scattering. J. Am. Chem. Soc..

[24] Campion A., Kambhampati P. (1998). Surface-Enhanced Raman Scattering. Chem. Soc. Rev..

[25] Jensen L., Aikens C.M., Schatz G.C. (2008). Electronic structure methods for studying surface-enhanced raman scattering. Chem. Soc. Rev..

[26] Morton S.M., Jensen L. (2009). Understanding the Molecule−Surface Chemical Coupling in SERS. J. Am. Chem. Soc..

[27] Haes A.J., Haynes C.L., McFarland A.D., Schatz G.C., Van Duyne R.P., Zou S. (2005). Plasmonic Materials for Surface-Enhanced Sensing and Spectroscopy. MRS Bull.

[28] Kleinman S.L., Frontiera R.R., Henry A., Dieringer J.A., Van Duyne R.P. (2013). Creating, Characterizing, and Controlling Chemistry with SERS Hot Spots. Phys. Chem. Chem. Phys..

[29] Le Ru E., Etchegoin P. (2008). Principles of Surface-Enhanced Raman Spectroscopy: And Related Plasmonic Effects.

[30] Kleinman S.L., Sharma B., Blaber M.G., Henry A., Valley N., Freeman R.G., Natan M.J., Schatz G.C., Van Duyne R.P. (2012). Structure Enhancement Factor Relationships in Single Gold Nanoantennas by Surface-Enhanced Raman Excitation Spectroscopy. J. Am. Chem. Soc..

[31] Greeneltch N.G., Blaber M.G., Henry A., Schatz G.C., Van Duyne R.P. (2013). Immobilized Nanorod Assemblies: Fabrication and Understanding of Large Area Surface-Enhanced Raman Spectroscopy Substrates. Anal. Chem..

[32] Kneipp K., Wang Y., Kneipp H., Perelman L.T., Itzkan I., Dasari R.R., Feld M.S. (1997). Single Molecule Detection using Surface-Enhanced Raman Scattering (SERS). Phys. Rev. Lett..

[33] Creighton J.A., Blatchford C.G., Albrecht M.G. (1979). Plasma resonance enhancement of Raman scattering by pyridine adsorbed on silver or gold sol particles of size comparable to the excitation wavelength. J. Chem. Soc. Faraday Trans. 2 Mol. Chem. Phys..

[34] Lee P., Meisel D. (1982). Adsorption and surface-enhanced raman of dyes on silver and gold sols. J. Phys. Chem..

[35] Xu H., Bjerneld E.J., Käll M., Börjesson L. (1999). Spectroscopy of single hemoglobin molecules by surface enhanced raman scattering. Phys. Rev. Lett..

[36] Xu H., Aizpurua J., Käll M., Apell P. (2000). Electromagnetic contributions to single-molecule sensitivity in surface-enhanced raman scattering. Phys. Rev. E.

[37] Rycenga M., Cobley C.M., Zeng J., Li W., Moran C.H., Zhang Q., Qin D., Xia Y. (2011). Controlling the synthesis and assembly of silver nanostructures for plasmonic applications. Chem. Rev..

[38] Lu X., Rycenga M., Skrabalak S.E., Wiley B., Xia Y. (2009). Chemical synthesis of novel plasmonic nanoparticles. Annu. Rev. Phys. Chem..

[39] Tian Z., Ren B., Wu D. (2002). Surface-enhanced raman scattering: From noble to transition metals and from rough surfaces to ordered nanostructures. J. Phys. Chem. B.

[40] Lal S., Grady N.K., Kundu J., Levin C.S., Lassiter J.B., Halas N.J. (2008). Tailoring plasmonic substrates for surface enhanced spectroscopies. Chem. Soc. Rev..

[41] Xu X., Hasan D., Wang L., Chakravarty S., Chen R.T., Fan D., Wang A.X. (2012). Guided-mode-resonance-coupled plasmonic-active SiO_2_ nanotubes for surface enhanced raman spectroscopy. Appl. Phys. Lett..

[42] Kim S., Zhang W., Cunningham B.T. (2008). Photonic crystals with SiO_2_-Ag “post-Cap” nanostructure coatings for surface enhanced Raman spectroscopy. Appl. Phys. Lett..

[43] Yan H., Gu C., Yang C., Liu J., Jin G., Zhang J., Hou L., Yao Y. (2006). Hollow core photonic crystal fiber surface-enhanced Raman probe. Appl. Phys. Lett..

[44] Perney N., de Abajo F.G., Baumberg J., Tang A., Netti M., Charlton M., Zoorob M. (2007). Tuning localized plasmon cavities for optimized surface-enhanced Raman scattering. Phys. Rev. B.

[45] Ackermann K.R., Henkel T., Popp J. (2007). Quantitative online detection of low-concentrated drugs via a SERS microfluidic system. Chem. Phys. Chem..

[46] Van Hyning D.L., Zukoski C.F. (1998). Formation Mechanisms and Aggregation Behavior of Borohydride Reduced Silver Particles. Langmuir.

[47] Ahern A.M., Garrell R.L. (1987). *In situ* photoreduced silver nitrate as a substrate for surface-enhanced raman spectroscopy. Anal. Chem..

[48] Frens G. (1972). Controlled Nucleation for the Regulation of the Particle Size in Monodisperse Gold Suspensions. Nature.

[49] Grabar K.C., Freeman R.G., Hommer M.B., Natan M.J. (1995). Preparation and Characterization of Au Colloid Monolayers. Anal. Chem..

[50] Amendola V., Meneghetti M. (2012). Exploring How to Increase the Brightness of Surface-Enhanced Raman Spectroscopy Nanolabels: The Effect of the Raman-Active Molecules and of the Label Size. Adv. Funct. Mater..

[51] Shaw C.P., Fan M.K., Lane C., Barry G., Jirasek A.I., Brolo A.G. (2013). Statistical correlation between SERS intensity and nanoparticle cluster size. J. Phys. Chem. C..

[52] Sant’Ana A.C., Rocha T.C. R., Santos P.S., Zanchetand D., Temperini M.L. A. (2009). Size-dependent SERS enhancement of colloidal silver nanoplates: the case of 2-amino-5-nitropyridine. J. Raman Spectrosc..

[53] Nie S., Emory S.R. (1997). Probing Single Molecules and Single Nanoparticles by Surface-Enhanced Raman Scattering. Science.

[54] Ni J., Lipert R.J., Dawson G.B., Porter M.D. (1999). Immunoassay Readout Method using Extrinsic Raman Labels Adsorbed on Immunogold Colloids. Anal. Chem..

[55] Cao Y.C., Jin R., Mirkin C.A. (2002). Nanoparticles with Raman Spectroscopic Fingerprints for DNA and RNA Detection. Science.

[56] Wang Y., Wei H., Li B., Ren W., Guo S., Dong S., Wang E. (2007). SERS Opens a New Way in Aptasensor for Protein Recognition with High Sensitivity and Selectivity. Chem. Commun..

[57] Amendola V., Scaramuzza S., Agnoli S., Polizzi S., Meneghetti M. (2014). Strong dependence of surface plasmon resonance and surface enhanced Raman scattering on the composition of Au–Fe nanoalloys. Nanoscale.

[58] Sun D.H., Zhang G.L., Jiang X.D., Huang J.L., Jing X.L., Zheng Y.M., He J., Li Q.B. (2014). Biogenic flower-shaped Au–Pd nanoparticles: Synthesis, SERS detection and catalysis towards benzyl alcohol oxidation. J. Mater. Chem. A.

[59] Lee Y.W., Kim N.H., Lee K.Y., Kwon K., Kim M.J., Han S.W. (2008). Synthesis and Characterization of Flower-Shaped Porous Au-Pd Alloy Nanoparticles. J. Phys. Chem. C..

[60] Li J.M., Yang Y., Qin D. (2014). Hollow nanocubes made of Ag–Au alloys for SERS detection with sensitivity of 10^−8^ M for melamine. J. Mater. Chem. C.

[61] Kong X., Yu Q., Zhang X., Du X., Gong H., Jiang H. (2012). Synthesis and Application of Surface Enhanced Raman Scattering (SERS) Tags of Ag@SiO_2_ Core/Shell Nanoparticles in Protein Detection. J. Mater. Chem..

[62] Liz-Marzán L.M., Giersig M., Mulvaney P. (1996). Synthesis of Nanosized Gold-Silica Core-Shell Particles. Langmuir.

[63] Álvarez-Puebla R.A., Contreras-Cáceres R., Pastoriza-Santos I., Pérez-Juste J., Liz-Marzán L.M. (2009). Au@pNIPAM Colloids as Molecular Traps for Surface-Enhanced, Spectroscopic, Ultra-Sensitive Analysis. Angew. Chem. Inter. Ed..

[64] Doering W.E., Nie S. (2003). Spectroscopic tags using dye-embedded nanoparticles and surface-enhanced raman scattering. Anal. Chem..

[65] Mulvaney S.P., Musick M.D., Keating C.D., Natan M.J. (2003). Glass-coated, analyte-tagged nanoparticles: A ‘new tagging system based on detection with surface-enhanced raman scattering. Langmuir.

[66] Kong X., Yu Q., Lv Z., Du X. (2013). Tandem assays of protein and glucose with functionalized core/shell particles based on magnetic separation and surface-enhanced raman scattering. Small.

[67] Li J.F., Huang Y.F., Ding Y., Yang Z.L., Li S.B., Zhou X.S., Fan F.R., Zhang W., Zhou Z.Y., Ren B. (2010). Shell-isolated nanoparticle-enhanced raman spectroscopy. Nature.

[68] Li J.F., Tian X.D., Li S.B., Anema J.R., Yang Z.L., Ding Y., Wu Y.F., Zeng Y.M., Chen Q.Z., Ren B. (2013). Surface analysis using shell-isolated nanoparticle-enhanced Raman spectroscopy. Nat. Protoc..

[69] Jun B., Noh M.S., Kim J., Kim G., Kang H., Kim M., Seo Y., Baek J., Kim J., Park J. (2010). Multifunctional silver-embedded magnetic nanoparticles as SERS nanoprobes and their applications. Small.

[70] Freeman R.G., Hommer M.B., Grabar K.C., Jackson M.A., Natan M.J. (1996). Ag-clad Au nanoparticles: Novel aggregation, optical, and surface-enhanced raman scattering properties. J. Phys. Chem..

[71] Shankar S.S., Rai A., Ahmad A., Sastry M. (2004). Rapid synthesis of Au, Ag, and bimetallic Au core–Ag Shell nanoparticles using Neem (Azadirachta Indica) leaf broth. J. Colloid Interface Sci..

[72] Akimov A., Mukherjee A., Yu C., Chang D., Zibrov A., Hemmer P., Park H., Lukin M. (2007). Generation of single optical plasmons in metallic nanowires coupled to quantum dots. Nature.

[73] Kumar G.P. (2012). Plasmonic nano-architectures for surface enhanced raman scattering: A review. J. Nanophotonics.

[74] Xia Y., Yang P., Sun Y., Wu Y., Mayers B., Gates B., Yin Y., Kim F., Yan H. (2003). One-dimensional Nanostructures: Synthesis, Characterization, and Applications. Adv. Mater..

[75] Tao A., Kim F., Hess C., Goldberger J., He R., Sun Y., Xia Y., Yang P. (2003). Langmuir-Blodgett Silver Nanowire Monolayers for Molecular Sensing using Surface-Enhanced Raman Spectroscopy. Nano Lett..

[76] Lee S.J., Morrill A.R., Moskovits M. (2006). Hot Spots in Silver Nanowire Bundles for Surface-Enhanced Raman Spectroscopy. J. Am. Chem. Soc..

[77] Link S., El-Sayed M.A. (1999). Spectral Properties and Relaxation Dynamics of Surface Plasmon Electronic Oscillations in Gold and Silver Nanodots and Nanorods. J. Phys. Chem. B.

[78] Nikoobakht B., El-Sayed M.A. (2003). Preparation and Growth Mechanism of Gold Nanorods (NRs) using Seed-Mediated Growth Method. Chem. Mater..

[79] von Maltzahn G., Centrone A., Park J., Ramanathan R., Sailor M.J., Hatton T.A., Bhatia S.N. (2009). SERS-coded Gold Nanorods as a Multifunctional Platform for Densely Multiplexed Near-infrared Imaging and Photothermal Heating. Adv. Mater..

[80] Wang Y., Yan B., Chen L. (2012). SERS Tags: Novel Optical Nanoprobes for Bioanalysis. Chem. Rev..

[81] Li W., Camargo P.H., Lu X., Xia Y. (2008). Dimers of Silver Nanospheres: Facile Synthesis and their use as Hot Spots for Surface-Enhanced Raman Scattering. Nano Lett..

[82] Wustholz K.L., Henry A., McMahon J.M., Freeman R.G., Valley N., Piotti M.E., Natan M.J., Schatz G.C., Duyne R.P.V. (2010). Structure−Activity Relationships in Gold Nanoparticle Dimers and Trimers for Surface-Enhanced Raman Spectroscopy. J. Am. Chem. Soc..

[83] Chen G., Wang Y., Yang M., Xu J., Goh S.J., Pan M., Chen H. (2010). Measuring Ensemble-Averaged Surface-Enhanced Raman Scattering in the Hotspots of Colloidal Nanoparticle Dimers and Trimers. J. Am. Chem. Soc..

[84] Lan X., Chen Z., Lu X., Dai G., Ni W., Wang Q. (2013). DNA-Directed Gold Nanodimers with Tailored Ensemble Surface-Enhanced Raman Scattering Properties. ACS Appl. Mater. Interfaces.

[85] Thacker V.V., Herrmann L.O., Sigle D.O., Zhang T., Liedl T., Baumberg J.J., Keyser U.F. (2014). DNA Origami Based Assembly of Gold Nanoparticle Dimers for Surface-Enhanced Raman Scattering. Nat. Commun..

[86] Novak J.P., Feldheim D.L. (2000). Assembly of Phenylacetylene-Bridged Silver and Gold Nanoparticle Arrays. J. Am. Chem. Soc..

[87] Sardar R., Heap T.B., Shumaker-Parry J.S. (2007). Versatile Solid Phase Synthesis of Gold Nanoparticle Dimers using an Asymmetric Functionalization Approach. J. Am. Chem. Soc..

[88] Ciou S., Cao Y., Huang H., Su D., Huang C. (2009). SERS Enhancement Factors Studies of Silver Nanoprism and Spherical Nanoparticle Colloids in the Presence of Bromide Ions. J. Phys. Chem. C.

[89] Yang Y., Zhong X., Zhang Q., Blackstad L.G., Fu Z., Li Z., Qin D. (2014). The Role of Etching in the Formation of Ag Nanoplates with Straight, Curved and Wavy Edges and Comparison of their SERS Properties. Small.

[90] Sun Y., Xia Y. (2002). Shape-Controlled Synthesis of Gold and Silver Nanoparticles. Science.

[91] McLellan J.M., Li Z., Siekkinen A.R., Xia Y. (2007). The SERS Activity of a Supported Ag Nanocube Strongly Depends on its Orientation Relative to Laser Polarization. Nano Lett..

[92] Barbosa S., Agrawal A., Rodríguez-Lorenzo L., Pastoriza-Santos I., Alvarez-Puebla R.A., Kornowski A., Weller H., Liz-Marzán L.M. (2010). Tuning Size and Sensing Properties in Colloidal Gold Nanostars. Langmuir.

[93] Hrelescu C., Sau T.K., Rogach A.L., Jäckel F., Feldmann J. (2009). Single Gold Nanostars Enhance Raman Scattering. Appl. Phys. Lett..

[94] Yuan H., Liu Y., Fales A.M., Li Y.L., Liu J., Vo-Dinh T. (2012). Quantitative Surface-Enhanced Resonant Raman Scattering Multiplexing of Biocompatible Gold Nanostars for *in Vitro* and *Ex Vivo* Detection. Anal. Chem..

[95] Xu P., Zhang B., Mack N.H., Doorn S.K., Han X., Wang H. (2010). Synthesis of Homogeneous Silver Nanosheet Assemblies for Surface Enhanced Raman Scattering Applications. J. Mater. Chem..

[96] Yan J., Han X., He J., Kang L., Zhang B., Du Y., Zhao H., Dong C., Wang H., Xu P. (2012). Highly Sensitive Surface-Enhanced Raman Spectroscopy (SERS) Platforms Based on Silver Nanostructures Fabricated on Polyaniline Membrane Surfaces. ACS Appl. Mater. Interfaces.

[97] Haynes C.L., Van Duyne R.P. (2001). Nanosphere Lithography: A Versatile Nanofabrication Tool for Studies of Size-Dependent Nanoparticle Optics. J. Phys. Chem. B.

[98] Hulteen J.C., Treichel D.A., Smith M.T., Duval M.L., Jensen T.R., Van Duyne R.P. (1999). Nanosphere Lithography: Size-Tunable Silver Nanoparticle and Surface Cluster Arrays. J. Phys. Chem. B.

[99] Zhang X., Yonzon C.R., Van Duyne R.P. (2006). Nanosphere Lithography Fabricated Plasmonic Materials and Their Applications. J. Mater. Res..

[100] Maxwell D.J., Emory S.R., Nie S. (2001). Nanostructured Thin-Film Materials with Surface-Enhanced Optical Properties. Chem. Mater..

[101] Dick L.A., McFarland A.D., Haynes C.L., Van Duyne R.P. (2002). Metal Film Over Nanosphere (MFON) Electrodes for Surface-Enhanced Raman Spectroscopy (SERS): Improvements in Surface Nanostructure Stability and Suppression of Irreversible Loss. J. Phys. Chem. B.

[102] Wang H., Levin C.S., Halas N.J. (2005). Nanosphere Arrays with Controlled Sub-10-Nm Gaps as Surface-Enhanced Raman Spectroscopy Substrates. J. Am. Chem. Soc..

[103] Qin L., Park S., Huang L., Mirkin C.A. (2005). On-Wire Lithography. Science.

[104] Qin L., Zou S., Xue C., Atkinson A., Schatz G.C., Mirkin C.A. (2006). Designing, Fabricating, and Imaging Raman Hot Spots. Proc. Natl. Acad. Sci. U. S. A..

[105] Banholzer M.J., Qin L., Millstone J.E., Osberg K.D., Mirkin C.A. (2009). On-Wire Lithography: Synthesis, Encoding and Biological Applications. Nat. Protoc..

[106] Chen X., Jeon Y., Jang J., Qin L., Huo F., Wei W., Mirkin C.A. (2008). On-Wire Lithography-Generated Molecule-Based Transport Junctions: A New Testbed for Molecular Electronics. J. Am. Chem. Soc..

[107] Qin L., Jang J., Huang L., Mirkin C.A. (2007). Sub-5-nm Gaps Prepared by On-Wire Lithography: Correlating Gap Size with Electrical Transport. Small.

[108] Xu X., Kwanoh K., Li H., Fan D. (2012). Ordered arrays of Raman nanosensors for ultrasensitive and location predictable biochemical detection. Adv. Mater..

[109] Xu X., Li H., Hasan D., Ruoff R.S., Wang A.X., Fan D. (2013). Near-Field Enhanced Plasmonic-Magnetic Bifunctional Nanotubes for Single Cell Bioanalysis. Adv. Funct. Mater..

[110] Fan D., Zhu F., Cammarata R., Chien C. (2011). Electric Tweezers. Nano Today.

[111] Muhlschlegel P., Eisler H.J., Martin O.J., Hecht B., Pohl D.W. (2005). Resonant Optical Antennas. Science.

[112] Bharadwaj P., Deutsch B., Novotny L. (2009). Optical Antennas. Advances in Optics and Photonics.

[113] Novotny L., Van Hulst N. (2011). Antennas for Light. Nat. Photonics.

[114] Taminiau T., Stefani F., Segerink F., Van Hulst N. (2008). Optical Antennas Direct Single-Molecule Emission. Nature Photonics.

[115] Etchegoin P.G., Le Ru E.C., Fainstein A. (2011). Bi-Analyte Single Molecule SERS Technique with Simultaneous Spatial Resolution. Phys. Chem. Chem. Phys..

[116] Fromm D.P., Sundaramurthy A., Kinkhabwala A., Schuck P.J., Kino G.S., Moerner W. (2006). Exploring the Chemical Enhancement for Surface-Enhanced Raman Scattering with Au Bowtie Nanoantennas. J. Chem. Phys..

[117] Jäckel F., Kinkhabwala A., Moerner W. (2007). Gold Bowtie Nanoantennas for Surface-Enhanced Raman Scattering Under Controlled Electrochemical Potential. Chem. Phys. Lett..

[118] Uda S. (1928). On the Wireless Beam of Short Electric Waves, by Shintaro Uda.

[119] Yagi H. (1928). Beam Transmission of Ultra Short Waves. Proc. Inst. Radio Eng..

[120] Crozier K.B., Zhu W., Wang D., Lin S., Best M.D., Camden J.P. (2014). Plasmonics for Surface Enhanced Raman Scattering: Nanoantennas for Single Molecules. IEEE J. Sel. Top. Quantum Electron..

[121] D’Andrea C., Bochterle J., Toma A., Huck C., Neubrech F., Messina E., Fazio B., Marago O.M., Di Fabrizio E., de La Chapelle L. (2013). Marc Optical Nanoantennas for Multiband Surface-Enhanced Infrared and Raman Spectroscopy. ACS Nano.

[122] Ward D.R., Grady N.K., Levin C.S., Halas N.J., Wu Y., Nordlander P., Natelson D. (2007). Electromigrated nanoscale gaps for surface-enhanced raman spectroscopy. Nano Lett..

[123] Diebold E.D., Peng P., Mazur E. (2009). Isolating Surface-Enhanced Raman Scattering Hot Spots using Multiphoton Lithography. J. Am. Chem. Soc..

[124] Buividas R., Stoddart P.R., Juodkazis S. (2012). Laser fabricated ripple substrates for surface-enhanced Raman scattering. Annalen Phys..

[125] Hu M., Ou F.S., Wu W., Naumov I., Li X., Bratkovsky A.M., Williams R.S., Li Z. (2010). Gold Nanofingers for Molecule Trapping and Detection. J. Am. Chem. Soc..

[126] Ou F.S., Hu M., Naumov I., Kim A., Wu W., Bratkovsky A.M., Li X., Williams R.S., Li Z. (2011). Hot-Spot Engineering in Polygonal Nanofinger Assemblies for Surface Enhanced Raman Spectroscopy. Nano Lett..

[127] Kim A., Ou F.S., Ohlberg D.A., Hu M., Williams R.S., Li Z. (2011). Study of Molecular Trapping Inside Gold Nanofinger Arrays on Surface-Enhanced Raman Substrates. J. Am. Chem. Soc..

[128] Kahl M., Voges E., Kostrewa S., Viets C., Hill W. (1998). Periodically Structured Metallic Substrates for SERS. Sens. Actuators B Chem..

[129] Kahl M., Voges E. (2000). Analysis of Plasmon Resonance and Surface-Enhanced Raman Scattering on Periodic Silver Structures. Phys. Rev. B.

[130] Dhawan A., Canva M., Vo-Dinh T. (2011). Narrow Groove Plasmonic Nano-Gratings for Surface Plasmon Resonance Sensing. Optics Express.

[131] Baltog I., Primeau N., Reinisch R., Coutaz J. (1995). Surface Enhanced Raman Scattering on Silver Grating: Optimized Antennalike Gain of the Stokes Signal of 104. Appl. Phys. Lett..

[132] Grande M., Bianco G., Vincenti M., Stomeo T., De Ceglia D., De Vittorio M., Petruzzelli V., Scalora M., Bruno G., D’Orazio A. (2012). Experimental Surface-Enhanced Raman Scattering Response of Two-Dimensional Finite Arrays of Gold Nanopatches. Appl. Phys. Lett..

[133] Jiao Y., Ryckman J.D., Ciesielski P.N., Escobar C.A., Jennings G.K., Weiss S.M. (2011). Patterned Nanoporous Gold as an Effective SERS Template. Nanotechnology.

[134] Ryckman J.D., Liscidini M., Sipe J., Weiss S. (2010). Direct Imprinting of Porous Substrates: A Rapid and Low-Cost Approach for Patterning Porous Nanomaterials. Nano Lett..

[135] Fan S., Joannopoulos J. (2002). Analysis of Guided Resonances in Photonic Crystal Slabs. Phys. Rev. B.

[136] Wang S., Magnusson R., Bagby J.S., Moharam M. (1990). Guided-Mode Resonances in Planar Dielectric-Layer Diffraction Gratings. JOSA A.

[137] Hu M., Fattal D., Li J., Li X., Li Z., Williams R.S. (2011). Optical properties of sub-wavelength dielectric gratings and their application for surface-enhanced raman scattering. Appl. Phys. A.

[138] Li J., Fattal D., Li Z. (2009). Plasmonic optical antennas on dielectric gratings with high field enhancement for surface enhanced raman spectroscopy. Appl. Phys. Lett..

[139] Kim S., Zhang W., Cunningham B.T. (2010). Coupling discrete metal nanoparticles to photonic crystal surface resonant modes and application to raman spectroscopy. Optics Express.

[140] Yang J., Ren F., Chong X., Fan D., Chakravarty S., Wang Z., Chen R.T., Wang A.X. (2014). Guided-Mode Resonance Grating with Self-Assembled Silver Nanoparticles for Surface-Enhanced Raman Scattering Spectroscopy. Photonics.

[141] Deep Blue Home. http://deepbluehome.blogspot.com/2011/01/psychedelic-diatoms.html.

[142] Jeffryes C., Campbell J., Li H., Jiao J., Rorrer G. (2011). The potential of diatom nanobiotechnology for applications in solar cells, batteries, and electroluminescent devices. Energy Environ. Sci..

[143] Song M., Park S., Alamgir F.M., Cho J., Liu M. (2011). Nanostructured electrodes for lithium-ion and lithium-air batteries: The latest developments, challenges, and perspectives. Mater. Sci. Eng. R Rep..

[144] Zhang Y., Liu G., Li H. (2006). Development of a micro swimming robot using optimised giant magnetostrictive thin films. Appl. Bionics Biomech..

[145] Jeffryes C., Solanki R., Rangineni Y., Wang W., Chang C., Rorrer G.L. (2008). Electroluminescence and photoluminescence from nanostructured diatom frustules containing metabolically inserted germanium. Adv. Mater..

[146] Qin T., Gutu T., Jiao J., Chang C., Rorrer G.L. (2008). Photoluminescence of silica nanostructures from bioreactor culture of marine diatom nitzschia frustulum. J. Nanosci. Nanotechnol..

[147] Losic D., Mitchell J.G., Lal R., Voelcker N.H. (2007). Rapid Fabrication of Micro- and Nanoscale Patterns by Replica Molding from Diatom Biosilica. Adv. Funct. Mater..

[148] Losic D., Rosengarten G., Mitchell J.G., Voelcker N.H. (2006). Pore Architecture of Diatom Frustules: Potential Nanostructured Membranes for Molecular and Particle Separations. J. Nanosci. Nanotechnol..

[149] Ren F., Campbell J., Wang X., Rorrer G.L., Wang A.X. (2013). Enhancing Surface Plasmon Resonances of Metallic Nanoparticles by Diatom Biosilica. Opt. Express.

[150] Ren F., Campbell J., Rorrer G.L., Wang A.X. (2014). Surface-Enhanced Raman Spectroscopy Sensors from Nanobiosilica with Self-Assembled Plasmonic Nanoparticles. IEEE J. Sel. Top. Quantum Electron..

[151] Yang J., Zhen L., Ren F., Campbell J., Rorrer G.L., Wang A.X. (2014). Ultra-sensitive Immunoassay Biosensors using Hybrid Plasmonic-biosilica Nanostructured Materials. J. Biophotonics.

[152] Stoddart P., White D. (2009). Optical Fibre SERS Sensors. Anal. Bioanal. Chem..

[153] Shi C., Zhang Y., Gu C., Chen B., Seballos L., Olson T., Zhang J.Z. (2009). Molecular Fiber Sensors Based on Surface Enhanced Raman Scattering (SERS). J. Nanosci. Nanotechnol..

[154] Stokes D.L., Vo-Dinh T. (2000). Development of an Integrated Single-Fiber SERS Sensor. Sens. Actuators B Chem..

[155] Smythe E.J., Dickey M.D., Bao J., Whitesides G.M., Capasso F. (2009). Optical Antenna Arrays on a Fiber Facet for in Situ Surface-Enhanced Raman Scattering Detection. Nano Lett..

[156] Kostovski G., Chinnasamy U., Jayawardhana S., Stoddart P.R., Mitchell A. (2011). Sub-15 nm Optical Fiber Nanoimprint Lithography: A Parallel, Self-aligned and Portable Approach. Adv. Mater..

[157] Xu W., Xu S., Lü Z., Chen L., Zhao B., Ozaki Y. (2004). Ultrasensitive Detection of 1, 4-Bis(4-Vinylpyridyl) Phenylene in a Small Volume of Low Refractive Index Liquid by Surface-Enhanced Raman Scattering-Active Light Waveguide. Appl. Spectrosc..

[158] Zhang Y., Gu C., Schwartzberg A., Zhang J. (2005). Surface-Enhanced Raman Scattering Sensor Based on D-Shaped Fiber. Appl. Phys. Lett..

[159] Yin D., Deamer D., Schmidt H., Barber J., Hawkins A. (2004). Integrated Optical Waveguides with Liquid Cores. Appl. Phys. Lett..

[160] Measor P., Seballos L., Yin D., Zhang J.Z., Lunt E.J., Hawkins A.R., Schmidt H. (2007). On-Chip Surface-Enhanced Raman Scattering Detection using Integrated Liquid-Core Waveguides. Appl. Phys. Lett..

[161] März A., Ackermann K.R., Malsch D., Bocklitz T., Henkel T., Popp J. (2009). Towards a Quantitative SERS Approach–online Monitoring of Analytes in a Microfluidic System with Isotope—edited Internal Standards. J. Biophotonics.

[162] Wang G., Lim C., Chen L., Chon H., Choo J., Hong J. (2009). Surface-Enhanced Raman Scattering in Nanoliter Droplets: Towards High-Sensitivity Detection of Mercury (II) Ions. Anal. Bioanal. Chem..

[163] Chen L., Choo J. (2008). Recent Advances in Surface—Enhanced Raman Scattering Detection Technology for Microfluidic Chips. Electrophoresis.

[164] Quang L.X., Lim C., Seong G.H., Choo J., Do K.J., Yoo S.K. (2008). A Portable Surface-Enhanced Raman Scattering Sensor Integrated with a Lab-on-a-Chip for Field Analysis. Lab Chip.

[165] Wang Y., Ni Z., Hu H., Hao Y., Wong C.P., Yu T., Thong J.T., Shen Z.X. (2010). Gold on Graphene as a Substrate for Surface Enhanced Raman Scattering Study. Appl. Phys. Lett..

[166] Xu W., Mao N., Zhang J. (2013). Graphene: A Platform for Surface—Enhanced Raman Spectroscopy. Small.

[167] West P.R., Ishii S., Naik G.V., Emani N.K., Shalaev V.M., Boltasseva A. (2010). Searching for Better Plasmonic Materials. Laser Photonics Rev..

[168] Naik G.V., Shalaev V.M., Boltasseva A. (2013). Alternative Plasmonic Materials: Beyond Gold and Silver. Adv Mater..

